# Vegetable as a Source of Bioactive Compounds with Photoprotective Properties: Implication in the Aging Process

**DOI:** 10.3390/nu15163594

**Published:** 2023-08-16

**Authors:** Justyna Moskwa, Monika Bronikowska, Katarzyna Socha, Renata Markiewicz-Żukowska

**Affiliations:** Department of Bromatology, Faculty of Pharmacy with the Division of Laboratory Medicine, Medical University of Białystok, 15-222 Białystok, Poland; monika75@op.pl (M.B.); katarzyna.socha@umb.edu.pl (K.S.); renmar@poczta.onet.pl (R.M.-Ż.)

**Keywords:** photoaging, skin protection, diet and UV protection, phytonutrients

## Abstract

The skin, as an external organ, protects the entire body against harmful external factors. One of these factors is ultraviolet (UV) radiation, which in excessive amounts can lead to premature skin aging, DNA damage, and even skin cancer. Therefore, it is worth supporting skin protection not only with commercially available preparations, but also with a proper diet. Consuming certain vegetables and applying them topically may reduce the effects of UV radiation. The aim of the review was to collect information on the effects of vegetables and their compounds on the skin when used externally or included in the diet. This review summarizes studies on vegetables, such as broccoli, cucumber, kale, tomato, and carrot, which have shown significant activity in skin photoprotection. Additionally, it outlines the bioactive substances present in these vegetables and their effects.

## 1. Introduction

Skin aging is a complex physiological phenomenon whose pathogenesis remains unclear despite extensive research. Understanding the mechanisms of aging is crucial in developing effective methods to delay this process. Among the various factors that accelerate skin aging, UV radiation is responsible for as much as 80% of its occurrence. Prolonged exposure to UV radiation not only leads to visible changes in the appearance of the skin, but also disrupts its function and structure [1]. During the course of photoaging, not only the appearance of the skin undergoes changes, but also its function and structure. Photoaging involves changes in the composition of the extracellular matrix and disorders in the functioning of the immune system. This is due to the suppression of the immune response and the induction of immunotolerance, potentially leading to skin carcinogenesis [2,3,4]. Ultraviolet radiation exposure upregulates matrix metalloproteinases (e.g., MMP1, MMP3, or MMP9) through the activation of the AP-1 pathway. This leads to the breakdown of collagen and elastin in the skin [5]. In addition, AP-1 activation inhibits the signaling of transforming growth factor (TGF)-β, which impairs the regeneration of collagen in the skin [5]. These changes contribute to skin thinning, the appearance of wrinkles, and dryness. Chronic exposure to UV can also lead to hypertrophic alterations, sebaceous gland hypertrophy, pigmentation disorders (hyperpigmentation, discoloration, and lentigine spots), as well as the development of telangiectasias [1,3,6,7].

Avoiding the sun on a daily basis is impossible; it is difficult to function normally and at the same time not expose yourself to UV radiation at all. However, it is worth resorting simple methods of protection, such as wearing a hat, long-sleeved clothing, or using an umbrella. Additionally, there is a wide range of pharmaceutical and cosmetic products available that contain sunscreens. It is also worth mentioning the natural mechanisms that humans have to protect their skin against UV rays.

Naturally, the human skin produces a protective filter through a process called melanogenesis. Melanin, a group of organic compounds, is responsible for skin pigmentation. Its primary function is to absorb UV radiation, thereby reducing the detrimental effects caused by radiation on melanocytes and keratinocytes. Melanocytes are pigment cells formed by melanogenesis from the neural crests. These cells produce melanin, which helps to diminish the penetration of UV radiation through the skin [8]. Melanosomes, organelles within melanocytes, transport melanin to keratinocytes, where a protective structure is formed to safeguard cell nuclei against the harmful effects of UV radiation [1]. This process provides protection to cells from folate damage and the breakdown of the upper layers of the skin, also reducing the risk of skin melanoma [4]. Nevertheless, the skin’s natural protection against UV is not sufficient, so it is necessary to support it.

Currently, the market offers a wide range of photoprotective preparations. The filters used in cosmetics can be divided into two groups: chemical and mineral (physical).

Chemical filters based on organic compounds, function by absorbing UV radiation. Examples of chemical filters include oxybenzone, avobenzone, and octocrylene. These aromatic compounds absorb high-intensity UV rays, which leads to their excitation to higher energy states. Subsequently, the absorbed energy is converted into lower energy waves, such as infrared radiation, and the molecules return to their ground state.

Chemical filters are widely used due to their effectiveness in providing UV protection. Moreover are often frequently chosen in view of the convenience they offer to users, because they do not cause a white cast on the skin [9,10,11]. Examples of mineral filters include zinc oxide and titanium dioxide. These white pigments protect the skin by reflecting or refracting UV radiation. Mineral filters are usually combined with chemical filters in sunscreen products. Although these pigments are effective filters, formulations that contain larger amounts of them cause noticeable whitening of the skin, which may be inconvenient for everyday use. To address this issue, is being used micronized form of physical filters or combination of physical and chemical filters is offering both high protection and improved cosmetic appearance. However, when used in micronized form with very small particles, their mechanism of action becomes similar to chemical filters. In this case, they behave like semiconductor metals that absorb UV across a wide range of the electromagnetic spectrum.

It is also worth mentioning that several natural raw materials support sun protection, e.g., raspberry seed oil [12], shea butter [13], or aloe vera [14]. While these may not ensure high levels of protection, they can serve as valuable additives to preparations that support photoprotection, at the same time nourishing and moisturizing the skin. These substances provide some defense against erythema, photoaging, and sunburn. Nevertheless, it is crucial to use the appropriate amount of product and reapply it regularly.

It is worth noting that external photoprotection is not the sole means of safeguarding oneself against UV damage. It is also possible to protect the skin by incorporating a healthy diet. The effect of the sun on the skin triggers oxidative mechanisms, leading to the production of singlet oxygen and free radicals. Numerous studies have confirmed the effectiveness of antioxidants in combating these harmful factors by neutralizing them through the donation of additional electrons, thereby preventing oxidative stress. Antioxidants naturally occur in plants, with examples including vitamins C and E, polyphenols, carotenoids (β-carotene, lutein, and lycopene), sulforaphane (SF), and certain minerals components, such as selenium, zinc, cobalt, copper, and manganese. Vegetables serve as an excellent source of the above nutrients. Vegetables are widely regarded as one of the healthiest food groups due to their abundant nutrient content and lower sugar content compared to fruit. They play an essential role in maintaining a healthy body, including the well-being of the skin, hair, and nails, and also support protection against UV radiation [15,16,17].

Given the significance of dietary factors and their impact on skin health, this review aims to provide an updated overview of the existing knowledge regarding the effects of specific vegetables, namely broccoli, cucumber, kale, tomato, and carrot, along with their bioactive ingredients (sulforaphane, lycopene, lutein, and β-carotene) on the protection of the skin against UV radiation. By examining the current state of research, this review sheds light on the potential benefits of incorporating these vegetables into the diet for enhancing skin protection.

**Figure 1:**
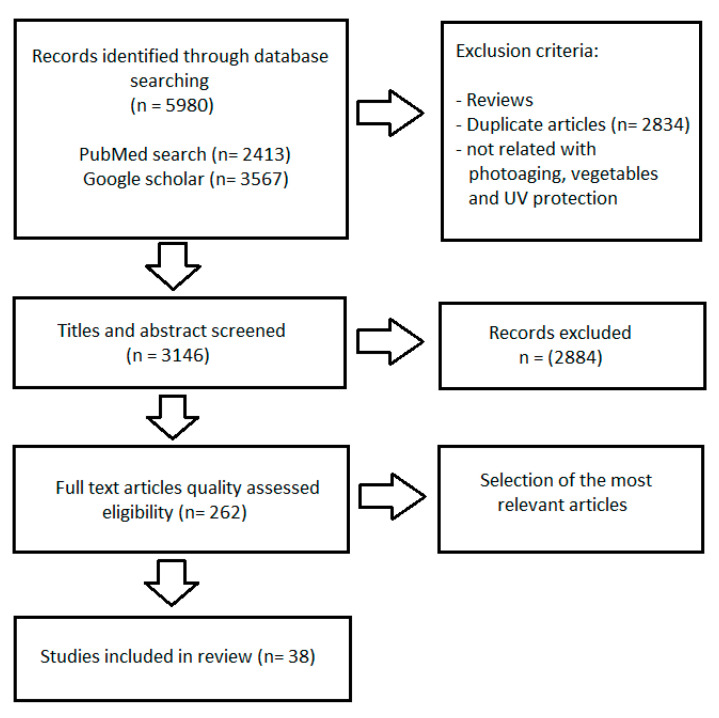
The literature search strategy and study selection criteria. UV: ultraviolet.

A comprehensive analysis was conducted on English literature available in the PubMed and Google databases spanning the years 2000–2023. The literature search included articles published until June 2023. The keywords employed in the search were as follows: “vegetables in photoprotection”, “vegetables and skin”, “natural photoprotection”, “skin protection against UV radiation”, “photoprotective filters”, “natural photoprotective filters”, “lycopene”, “lycopene and skin”, “tomato in photoprotection”, “tomato UV protection”, “sulforaphane”, “sulforaphane and skin”, “sulforaphane in photoprotection”, “sulforaphane UV protection”, “broccoli in photoprotection”, “kale and skin”, “kale in photoprotection”, “kale UV protection”, “lutein”, “lutein and skin”, “lutein in photoprotection”, “beta carotene”, “beta carotene and skin”, “beta carotene and photoprotection”, “carrot in photoprotection”, “carrot UV protection”, “cucumber and skin”, “cucumber in photoprotection”, “cucumber UV protection”, and “diet and healthy skin”. There were identified 5980 studies in the searched databases. Among them 262 articles were qualified after selection procedure and finally, 38 were included in this manuscript (Figure 1).

## 2. The Skin as the First Protective Barrier against UV Radiation

The skin, being the largest organ of the human body and constituting approximately 16% of body weight, plays a crucial part as a protective barrier. It acts as a shield between the body and the external environment. The stratum corneum is particularly vulnerable to external threats, having direct contact with infectious pathogens, physical and chemical factors, and being exposed to UV radiation. Therefore, it is of utmost importance to prioritize the care and protection of the skin from external factors [2,8,18]. Ultraviolet radiation can originate from both artificial and natural sources. The former include lamps used for polymer curing, tanning beds, mercury lamps, halogen lights, germicidal lamps, as well as some types of laser, such as nitrogen, excimer, and third harmonic (Nd:YAG) lasers. On the other hand, the sun serves as the natural source of UV, accounting for approximately 7% of the radiation that reaches our planet. UV radiation is classified into three types based on wavelength. UVA radiation has the longest wavelength (320–400 nm), but consists of the least energetic photons. UVB radiation falls into the medium wavelength range (280–320 nm) and possesses medium energy radiation. UVC, with the shortest wavelength, has the highest energy level [6].

Nearly all UVC radiation and 90% of UVB emissions are absorbed by the Earth’s atmosphere containing oxygen and ozone. As a result, these types of radiation have limited impact on our exposure. Conversely, UVA radiation constitutes the highest proportion of UV radiation that reaches our planet, accounting for approximately 96.99% of the total. UVB radiation, while less prevalent, still contributes to our overall UV exposure, making up approximately 1–4% of the radiation [3].

**Figure 2:**
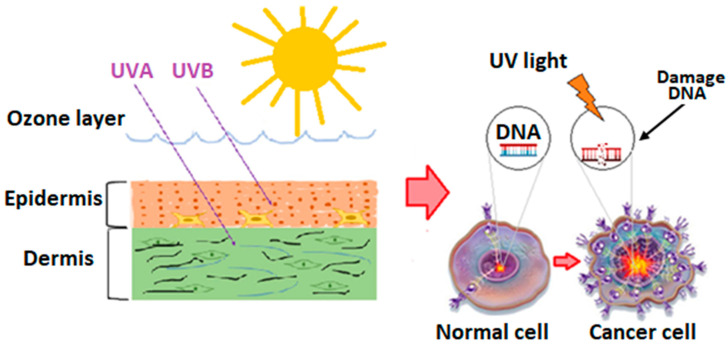
Effect of UV radiation on human skin. UV: ultraviolet; UVA: ultraviolet A; UVB: ultraviolet B.

UV radiation poses a significant risk as it can penetrate not only the outermost layer of the skin (epidermis), but also reach into the dermis (Figure 2). Moreover, 20–50% of the radiation can reach melanocytes [7]. Specifically, UVB extends to the deep layers of the epidermis and can, to a limited extent, reach the upper layers of the dermis. Approximately 9–15% of UVB radiation infiltrates the level of melanocytes [7]. Theoretically, UVC radiation is not considered a threat since it is practically completely absorbed by the atmosphere, including the protective ozone layer.

UV radiation is absorbed by various endogenous chromophores present in the skin, e.g., nucleic acid, aromatic amino acids, and acids and precursors found in the epidermis. Deoxyribonucleic acid (DNA) absorbs four times more UVB than UVA. This increased absorption can initiate carcinogenic processes and cell mutations. UV radiation is also absorbed by tyrosine and tryptophan. When these amino acids bind to DNA, they can cause photodamage. Additionally, proteins containing amino acids can be susceptible to damage from UV radiation [2,7]. Other detrimental effects of UV radiation include telangiectasia, the formation of free radicals, and oxidative stress.

### 2.1. Generation of Reactive Oxygen Species in Skin Aging

UV radiation is a harmful factor that triggers the generation of reactive oxygen species (ROS), which are highly reactive atoms with unpaired electrons. In an effort to achieve homeostasis, ROS attack healthy surrounding structures, seeking to acquire atoms from them. As a result, oxidation reactions occur, leading to damage in proteins, lipids, and even the DNA of human cells. The reason for this mechanism is UVA radiation, which leads to the formation of ROS. UVA is absorbed by intracellular photosensitizers, such as porphyrins, melanin, and flavoproteins or exogenous sensitizing substances, e.g., immunosuppressive drugs [19]. ROS encompass various entities, for instance, the superoxide anion, hydrogen peroxide, and the hydroxyl radical. ROS cause e.g., activation of metalloproteinases, as a result of which collagen fibers are damaged, resulting in loss of skin firmness. Moreover, the destructive effects of ROS on proteins and enzymes can inhibit cell proliferation and cause various abnormalities in their functioning. It is ROS, acting as mediators of UV radiation, that are the primary factor in accelerating skin aging. The consequences of ROS action on the skin are evident in structural changes. The epidermis undergoes thinning, as a result of atrophy of the granular and spinous layers. In the dermis, collagen and elastin fibers progressively diminish, which leads to a loss of skin firmness and elasticity.

ROS also cause damage to nucleotides where DNA damage occurs. This process can generate mutagenesis or even lead to cell death [20,21]. One example of a gene point mutation is the conversion of guanine to thymine, which occurs through oxidation of guanine at the 8th position, producing 8-hydroxy-2′-deoxyguanosine (8-OHdG). The action of free radicals leads to this mutation. Interestingly, 8-OHdG has a tendency to bind to adenine instead of cytosine, which suggests that it is the action of ROS that causes the mutation of the G/C pair into an A/T pair. This specific mutation highlights how ROS-induced damage can result in gene mutations and even contribute to tumor formation [2,21].

### 2.2. Skin Cancers

The most concerning complication of chronic UV exposure is the development of skin cancer. Absorption of large doses of radiation over a short period of time significantly increases the risk of cancer. UV radiation is known to contribute to the development of melanoma as well as non-melanoma skin cancers, such as basal cell carcinoma or squamous cell carcinoma. In the United States, the incidence of skin cancer in 2019 was 30.9% per 100,000 inhabitants in men and 18.4% per 100,000 in women [22]. It is estimated that in 2023, there will be approximately 57,120 new cases of cancer in men and 39,490 in women in the United States alone [15]. These statistics emphasize the significance of the effects of UV radiation as a health problem.

UVB radiation has a much greater genotoxic effect compared to UVA. When exposed to direct UVB emission, DNA absorbs the radiation, leading to the formation of cyclobutane pyrimidine dimers and pyrimidine(6-4) pyrimidone photoproducts. Typically, dimerization occurs between two thymines (5′-TT-3′), but various combinations of pyrimidines are possible, such as 5′-CT-3′, 5′-TC-3′, and 5′-CC-3′ [19]. This process can result in genetic abnormalities that disturb the mechanisms of cell growth and differentiation, impairing their proper functioning. Accumulation of multiple mutations in genes can transform a cell into a cancer cell. Tumor formation involves three stages: initiation, promotion, and progression, each driven by new mutations in different groups of genes. The activation of proto-oncogenes and the inactivation of anti-oncogenes play a significant role in the process of tumorigenesis. Subsequent mutations of DNA repair genes: suppressor genes during the metastasis process, involved in the apoptosis and angiogenesis pathways, which regulate cell adhesion, genes for immunoglobulin cytokines and leukocytes also contribute to the formation of tumors and their spread [19]. These gene mutations demonstrate that excessive exposure to UV radiation has an adverse effect not only on the skin but also on the entire body. In the case of cancer development, it can pose a significant threat to life.

## 3. Vegetables as a Source of Photoprotective Ingredients

Vegetables are a highly valuable food group that provides essential nutrients for overall health, including the skin. They are rich in vitamins, dietary fiber, and minerals. Vegetables are a natural source of well-absorbed vitamins, including A, C, K, and B-group vitamins, as well as polyphenols. Particularly valuable for the skin are polyphenols, including sulforaphane (SF) and carotenoids, such as β-carotene, lutein, and lycopene. Research has shown that the components found in vegetables have the potential to prevent premature signs of skin aging and offer protection against UV radiation. Numerous skincare products in the market utilize vegetable extracts as effective active ingredients. Interestingly, consuming vegetables can also help mitigate the side effects of UV radiation on the skin. Vegetables that deserve special mention are: broccoli, cucumber, kale, tomato, and carrot.

### 3.1. Broccoli

Broccoli (*Brassica oleracea* var. *italica Plenck*), a vegetable of the Brassicaceae family, has a long history of consumption, dating back to ancient Greece and Rome. Today, it remains a popular vegetable, widely used in the kitchen, appreciated for its taste and nutritional value. In the United States, the per capita consumption of broccoli was recorded at 6.47 g per day in 2021 [23]. Broccoli contains many important bioactive components, such as antioxidants glucoraphanin, flavonoids (quercetin, kaempferol), vitamins (C, A, and B-group), carotenoids (lutein), and minerals (e.g., potassium and selenium) [24,25,26]. The nutrients present in broccoli contribute to its potential in skincare, both when applied topically and when included in the diet [27,28,29,30]. Among the best studied compounds contained in broccoli is SF (degradation product of glucoraphanin), which has demonstrated effects in mitigating the negative effects of UV radiation [31,32]. Additionally, SF has been found to reduce susceptibility to erythema [33]. The protective effect of SF against the formation of free radicals and its potential effect against skin cancer have been confirmed by many authors [27,28,29,34,35].

Research has demonstrated that lutein found in broccoli can effectively prevent the formation of wrinkles caused by UVB radiation [30], while quercetin has been found to prevent collagen degradation [36]. Moreover, kaempferol, present in broccoli and known for its antioxidant, anti-inflammatory, and anti-cancer properties [37], can effectively support sun protection [38,39]. Apart from its nutritional properties and ability to protect the skin against the negative effects of UV radiation, it is worth noting that broccoli juice may also be beneficial in the treatment of warts [26].

### 3.2. Cucumber

Cucumber (*Cucumis sativus* L.) belongs to the Cucurbitaceae family and is widely consumed worldwide, either raw or in the form of salads or pickles [40]. In Ayurvedic medicine, cucumber leaves, fruits and seeds are valued for their potential in the prevention of skin aging [41,42]. Cucumbers are also used to relieve symptoms of skin problems such as sunburn as they have a cooling, soothing, and softening effect when applied topically. Cucumber fruits are composed of approximately 96.4% water, 2.8% carbohydrates, 0.4% protein, 0.1% fat, and 0.3% minerals, including 0.01% calcium, 0.03% phosphorus, and 0.0015% iron [40]. They also contain various enzymes, such as proteases, ascorbate oxidases, succinic dehydrogenase, and malate dehydrogenase. In addition, cucumbers contain ascorbic acid [41], and the extract from its pulp and peel has been found to contain lactic acid (7–8% *w*/*w*) with antioxidant properties [43]. A study by Gill et al. (2009) demonstrated that the methanol extract solution of cucumber seeds, compared to chloroform, ethyl acetate, and methanol extracts, showed antioxidant and photoprotective potential [44]. Furthermore, lutein present in cucumber leaves may contribute to inhibiting melanin synthesis and reducing the expression of tyrosinase. Kai et al. (2008) reported that the MeOH extract from cucumber leaves, when applied topically to B16 mouse melanoma cells, demonstrated potential in photoprotection not only against UVA and UVB radiation but also through its antioxidant activity [45].

### 3.3. Kale

Kale (*Brassica oleracea* L. var. *Acephala*) has been cultivated for centuries, but its popularity soared after 2010 in the United States. Kale is a popular vegetable and is often referred to as a “superfood” due to its high nutritional value. Kale leaves are commonly consumed raw in salads or juiced. They can also be cooked and used in soups, omelettes, or stir-fries. Belonging to the cruciferous family, kale possesses rich nutritional properties. It contains various phytochemicals, including polyphenols, vitamins C, E and A, SF, lutein, and β-carotene [46,47]. Sulphorafane, in particular, is a well-studied flavonoid that has demonstrated protection against UV radiation and a preventive effect against skin cancer [27,28,34]. Lycopene and lutein, both from the carotenoid group, are other powerful antioxidants. Lycopene protects the skin from harmful UV radiation and even prevents cancer [48,49], and lutein helps prevent the skin from dehydration and premature aging [35,50]. Regular consumption of kale can contribute to slowing down skin aging and reducing skin thinning caused by UV radiation exposure [51]. Kale is also a rich source of antioxidants, which indicates its potential effectiveness in combating free radicals [52]. Meinke et al. (2017) conducted a clinical study in which volunteers were supplemented with a carotenoid-rich natural kale extract containing a total of 1650 µg of carotenoids. The results showed significantly increased values of dermal carotenoids and the collagen I/elastin aging index at 5 and 10 months after the start of the study. Which means that the natural extract rich in carotenoids can prevent aging-related degradation of collagen I in the dermis and improve the extracellular matrix [53]. Although there is currently limited research in this specific area, it is important to stay updated on advancements in research regarding kale’s role in preventing the harmful effects of UV radiation.

### 3.4. Tomato

Tomato (*Solanum lycopersicum* L., *Lycopersicon esculentum* Mill.), a vegetable native to Peru, South America, belongs to the nightshade family and is widely known and consumed worldwide. It is enjoyed both raw in salads and cooked in sauces and soups. It is a popular vegetable due to its taste and nutritional properties. Tomatoes are rich in various nutrients, including vitamins (C and E), minerals (potassium), proteins, carotenoids (lycopene and β-carotene), phytosterols (β-sitosterol, campesterol, and stigmasterol), and phenolic compounds (kaempferol, quercetin, lutein, ferulic acid, chlorogenic acid, and caffeic acid) [54,55]. These compounds, particularly antioxidants, have a considerable impact on the condition of the skin and may prevent aging and photoaging. A number of studies have highlighted lycopene as a potent antioxidant. It protects the skin against the harmful effects of UV radiation, reduces inflammation, prevents DNA damage, and even decreases the number of tumors [49,50]. Research has shown that consuming tomato paste (40 g per day, containing 16 mg of lycopene) elevates the levels of carotenoids in the skin and plasma and reduces inflammation caused by UVB radiation. Topical application and including tomatoes in the diet before UV exposure can lower the risk of cancerous tumors during a person’s lifetime [49,56].

### 3.5. Carrot

Carrot (*Daucus carota* L.), the most popular root vegetable, belongs to the celery family and originates from Asia [57]. It is a staple of many cuisines, commonly used in salads, soups, sauces, and preserves. Carrots have a diverse nutrient profile, including vitamins A, C, E, K, H, and B-group vitamins. They also contain an array of minerals: calcium, potassium, sodium, iron, copper, phosphorus, magnesium, zinc, and cobalt. Additionally, carrots provide folic acid, malic acid, inositol, and pectin. Carrots are also rich in carotenoids: β-carotene (6.15–9.02 mg/100 g)—provitamin A, α-carotene (0.53–4.96 mg/100 g), lutein and zeaxanthin (0.30–0.51 mg/100 g), and small amounts of lycopene (0.015 mg/100 g) [58]. Furthermore, luteolin present in carrots may be effective in preventing photoaging of the skin [59]. Carrot seed oil has been recognized for its great potential as an ingredient in anti-aging cosmetics thanks to its antioxidant properties and SPF protection of 6.92 [60]. In addition, research confirms that the methanol extract derived from the powdered cell line (R4G) of red carrot (*Daucus carota* L.) is abundant in anthocyanins, which possess strong antioxidant and anti-inflammatory properties and thus have a high potential for preventing skin aging [61].

## 4. Photoprotective Bioactive Food Ingredients

Vegetables are rich sources of vitamins, minerals, and polyphenols all of which have a beneficial impact on the condition of the skin. However, this review also emphasizes the significance of ingredients such as SF, lycopene, luteolin, and β-carotene in their ability to protect against the negative effects of UV radiation, whether applied topically or consumed with the diet. The photoprotection effect of natural extracts form vegetables and their active compounds has been summarized in Table 1 and Table 2.

### 4.1. Glucoraphanin

Glucoraphanin, an organosulfur compound from the group of isothiocyanates, found in cruciferous vegetables serves as a natural defense mechanism to deter predators while exhibiting selective antibiotic properties [69]. Broccoli and its sprouts are particularly rich sources of glucoraphanin. This compound acts as a precursor to sulforaphane (SF) [28], which has been shown to protect the skin from UV-induced carcinogenesis such as non-melanoma skin cancer [28,34]. Topical application of broccoli sprout extract containing SF can prevent skin aging and alleviate sun pigmentation [31].

Dinkova-Kostova et al., between 2006 and 2010, conducted a series of studies on the effects of topically applied and dietary SF on both mice and humans. In one of their initial studies, they observed that when applied topically (on SKH-1 hairless mice), SF led to a reduction of up to 50% in the number and size of tumors [27]. The mice were irradiated with UVB (30 mJ/cm^2^/session) twice a week for 20 weeks and then divided into three groups. The control group received topical treatment with 100 mL of 80% acetone, while the other two study groups were treated with 100 mL of broccoli sprout extract containing either 0.3 mmol of SF or 1.0 mmol of SF. The animals received treatment 5 days a week for 11 weeks. It was observed that by the end of the study, all mice in the control group had developed at least one tumor. Additionally, human and mouse primary epidermal (PE) keratinocytes were tested with either 1 μM or 5 μM of SF and then exposed to UV irradiation [27]. Another study by Dinkova-Kostova et al. reported that SF had the potential to prevent the development of skin carcinogenesis [29]. The study was performed on two groups of hairless SKH-1 mice, which were then sacrificed and evaluated histologically. In addition, a study was conducted on human volunteers, where SF (from broccoli sprout extract) was topically applied, followed by a skin biopsy. The biopsy confirmed an increase in the enzymatic activity of NQO1 in the skin [29]. In the last research study conducted by Dinkova-Kostova et al. (2010) hairless SKH-1 mice were subjected to chronic UV exposure (30 mJ/cm^2^ of UVB, twice a week, for 17 weeks), followed by a diet supplemented with broccoli sprout extract (with a daily dose of 10 µmol of glucoraphanin). It was observed that tumor growth was inhibited for the subsequent 13 weeks. The number and size of tumors decreased by 25%, 47%, and 70%, respectively, in mice that received the broccoli extract compared to the control group [28]. Broccoli sprout extract showed a cytoprotective effect when applied both topically and orally [27,28]

In a study conducted by other authors, seven-eight-week-old Swiss albino mice were divided into five study groups, including one control group. The mice were treated with either acetone or dimethylbenz(a)anthracene (DMBA) in acetone on shaved skin and SF was administered orally at a dosage of 9 μmol/mouse/day. The study revealed that SF attenuated the activation of inflammatory and apoptotic pathways induced by skin cancer. Additionally, SF inhibited skin cancer development by blocking sulfatase-2 [35]. Benedict et al. (ROK) conducted a study involving primary SKH-1 dermal fibroblasts, or keratinocytes. The cells were treated with a solvent (0.1% acetonitrile) or 1 μM of SF for 24 h and then exposed to 20 mJ/cm^2^ of broadband UVB radiation. The results showed that SF did not affect the formation of UVB-induced DNA photoproducts. In the next part of the study, the cells were also treated with a solvent (0.1% acetonitrile) or 1 μM of SF for 24 h and then exposed to UV radiation. Control cells were shielded from the radiation (wrapped in foil) but exposed to UV lamps for the same duration as the highest dose of UV radiation. The study showed that SF induced the expression of NQO1 and provided protection against the formation of ROS caused by UV radiation [62]. Talalay et al. (2007) conducted a study where SKH-1 mice were topically treated with an extract containing 0.5 μmol of SF in 50 μL of a solution consisting of 80% acetone/20% water (*v*/*v*) derived from broccoli sprouts in three doses. The mice were then sequentially exposed to 700 mJ/cm^2^ 311-nm UVR. A human study was also conducted, involving subjects with skin phototypes 1 (always burns, never tans), 2 (always burns, sometimes tans), or 3 (sometimes burns, always tans). Male volunteers received 100, 200, 400, or 600 nmol of SF in 25 μL of a solution consisting of 80% acetone/20% water, or 200 nmol SF in 25 μL of the same solution, or solvent alone for 3 days at 24-h intervals. Narrowband UV (311 nm) was applied to the human subjects as part of the experiment. In addition, six men and three women who were exposed to UV (311 nm) were treated with 25 μL of an extract containing 200–400 nmol of SF in 80% acetone/20% water, while a control group received only 25 μL of the solvent. The study showed that 3-day-old broccoli sprout extract upregulated phase 2 enzymes in both mouse and human skin, was able to protect against UVR-induced inflammation and edema in mice, and reduced susceptibility to erythema (311-nm UVR) in humans [33]. Kerns et al. (2021) demonstrated that SF derived from broccoli significantly increased the level of NRF2 in 6 out of 8 people tested where induction with UV radiation occurred. NRF2 acts as an antioxidant and its signaling is dysregulated in stain lentigines. In addition, compared to the control group, there was an approximate 50% reduction in melanin and a 30% reduction in the expression of tyrosinase. A study conducted in K14-Cre-ERT2IL-6Rα/mice showed that the SF compound did not prevent UV-induced hyperpigmentation when applied topically. This suggests that the prevention of hyperpigmentation may be dependent on NRF2. In contrast, in a study involving male C57BL/6 mice exposed to UVB radiation, sequential topical application of SF to the right ear (UVB + SF) was found to alleviate UV-induced discoloration compared to the group where the left ear was treated with oil (UVB + Oil), or the group where UVB was not applied [31]. Chawalitpong et al. (2019) confirmed that glucoraphanin-enriched kale (GEK) fed at a 1% concentration in a standard diet inhibited aging in SAMP1 mice. This effect was achieved by increasing antioxidant activity and collagen production via the TβRII/Smad3 pathway [53].

It is also worth mentioning the study on synthetic SF, which has a protective effect against sunburn caused by UV radiation exposure. This has been showed by Saw et al. in studies on mice, where inflammation was induced by UVB lamps (300 mJ/cm^2^. NRF2 KO and WT C57BL mice were divided into six groups and treated topically with acetone or synthetic SF. Histological examination confirmed that SF mitigated the effects of UV radiation and exerted a protective effect on the skin through NRF2, restoring the thickness of the skin to its normal level [32].

### 4.2. Lycopene

Lycopene is a polyunsaturated hydrocarbon and a potent antioxidant belonging to the carotenoid family. It is found in vegetables and fruits, with red tomatoes and tomato products being the richest sources. It is also present in carrots, pumpkin, peppers, and sweet potatoes. In the United States, tomatoes contribute to as much as 80% of the lycopene consumed. The concentration of lycopene in tomatoes can vary depending on the variety, with red tomatoes containing up to 50 mg per kg and yellow ones containing only 5 mg per kg. It is also worth mentioning that the bioavailability of lycopene and other carotenoids can be enhanced through thermal processing. Heat treatment helps release lycopene from the food matrix, facilitating the absorption of lipophilic compounds to form lipid micelles together with dietary lipids and bile acids [59].

Fazekas et al. (2003) conducted a study that demonstrated the strong protective effect of lycopene against photodamage caused by UVB radiation. The experiment involved 6–7 week old SKH-1 mice, divided into four groups. Group 1 received apparent radiation, Group 2 was exposed to UVB only, Group 3 received topical application of 0.05 qmol of lycopene + UVB, and Group 4 received topical application of 0.1 qmol of lycopene + UVB. The findings indicated that lycopene prevented inflammatory reactions, interfered with the apoptosis pathway by inhibiting caspase-3, and had the potential to inhibit the activity of epidermal ornithine decarboxylase [50]. Rizwan et al. (2011) conducted a study on 20 white women with phototypes I and II, aged 21–47. They were divided into two groups. Group 1 received a diet supplemented with tomato paste and olive oil, while Group 2 served as the control group and received only olive oil. The daily dose of tomato paste was 55 g, which provided 16 mg of lycopene, served together with 10 g of olive oil on white bread. Subsequently, the women were exposed to UVR radiation with an emission range of 270–400 nm. The findings of this study confirmed that lycopene exerted a protective influence against UVR-induced damage, specifically in relation to mtDNA and MMP-1 deletions. This protective effect was attributed to lycopene’s ability to minimize ROS and oxidative stress. Lycopene was found to influence cell signaling and exhibit antioxidant activity, thus protecting tissues from the harmful impact of radiation [65]. In a study by Ascenso et al. (2015), cells were exposed to UVB lamps with a peak emission of 312 nm. The control group was exposed to radiation alone, while the test group was exposed to complexed lycopene at a concentration of 10 μM prior to UVB irradiation. In UV-exposed cells, lycopene treatment induced overexpression of the BAX gene compared to cells without irradiation. This led to a delay in the cell cycle at the S-phase transition, resulting in a decrease in the number of cells in the G0/G1 phase. This means that lycopene may have regenerative effects on irradiated cells, potentially contributing to the repair of photodamage caused by UV radiation [70]. Andreassi at al. (2004) conducted a study where 10 healthy volunteers (women and men, 20–42 years old, phototypes II and III) had gel formulations applied to their forearms. One group received gel with lycopene (at a concentration of 3 mg/cm^2^), while another one received gel with vitamins C and E. After 30 min, the treated areas were irradiated with a UV lamp. The study found that the lycopene-based product provided better protection, which confirms that it is a valuable antioxidant [71]. Stahl et al. (2001) carried out a study on 8 men and 14 women aged 26–67 (phototype II). The control group received 10 g of olive oil per day, while the study group received 40 g of tomato puree (containing 16 mg of lycopene, 0.5 mg of β-carotene, and 0.1 g of lutein) along with 10 g of olive oil per day. It was found that the test group that consumed tomato paste had a 40% higher protection against UV-induced erythema compared to the control group, as measured at week 10 [56]. Cooperstone et al. (2017) conducted an experiment where four-week-old male and female SKH-1 hairless mice were fed a diet containing 10% lycopene from powdered tomatoes for 35 weeks. From the 11th to the 20th week, the skin on the backs of the mice was exposed to UVB radiation at a dose of 2240 J/m^2^. The mice fed the lycopene diet had significantly fewer tumors compared to the control group. This suggests that a diet enriched with lycopene may help reduce the risk of developing cancer [49].

A study conducted by Aust et al. (2005) involved 36 healthy volunteers with phototype II. Minimal controlled erythema was induced in the participants, and they were divided into three groups. The first group took 2 capsules containing 5.1 mg/capsule of lycopene. The second group received a tomato extract capsule containing 4.9 mg of lycopene, 0.4 mg of phytofluene, 0.5 mg of phytoene, and 0.2 mg of β-carotene. The third one consumed 250 mL of a solubilized drink twice a day (4.1 mg of lycopene, 1.6 mg of phytofluene, 2.3 mg of phytoene, and 0.2 mg of β-carotene). At 4 and 12 weeks, erythema was induced again. The group consuming the solubilized drink achieved the best results, with a 48% reduction in erythema compared to the initial level, followed by the group consuming the tomato extract (38% reduction), and the synthetic lycopene group (25% reduction). Lycopene from tomato extract produced better results than synthetic lycopene. This could be attributed to the presence of other carotenoids such as phytofluene and phytoene, which have absorption maxima at UV exposure. This study proves that the type of lycopene is significant, and the best protection is provided by natural sources [66]. Stahl et al. (2006) conducted an experiment with humans who consumed different lycopene sources: 40 g of tomato puree + 10 g of olive oil (providing 16 mg of lycopene per day, for 10 weeks), 2 × 200 mL of carrot juice (providing 10 mg of lycopene per day and 5.1 mg of β-carotene for 12 weeks), a lycopene supplement with tomato juice extract (providing 9.8 mg of lycopene and 0.5 mg of β-carotene per day for 12 weeks), 2 × 250 mL of lycopene drink from tomato extract (providing 8.2 mg of lycopene and 0.4 mg of β-carotene per day for 12 weeks), or synthetic lycopene (10.2 mg per day). After 12 weeks of lycopene supplementation, the subjects were exposed to UV radiation, and the best photoprotective results were observed with the lycopene drink, resulting in a 50% decrease in erythema. Carrot juice showed a 45% decrease, while tomato puree showed a 40% decrease [59]. These findings also highlight the importance of the source of lycopene in providing photoprotection. Austa et al. and Stahl et al. both demonstrated that synthetic lycopene had a much lower protective effect than lycopene of natural origin [59,66].

### 4.3. Luteolin

Luteolin is a bioactive compound that can be found in spinach, kale, onion, broccoli, celery, corn, and carrot. The recommended daily intake of luteolin is around 10.0 mg, but studies have shown that the average daily consumption of luteolin in the USA is only about 1.7 mg per day [72]. Luteolin acts as a high-energy UV filter and is also a potent antioxidant. In addition to protecting the skin against UV damage, it has been confirmed that luteolin may have benefits in delaying the onset of eye diseases, for example, age-related macular degeneration (AMD) and cataracts [73].

In a study conducted by Lim et al. (2013) on hairless 5-week-old SKH-1 mice, the mice were divided into 4 groups. The control group received topical application of 200 μL of acetone, while the UVB group received topical application of 200 μL of acetone 1 h before UVB (0.18 J/cm^2^) irradiation. The other two groups were given topical luteolin (10 or 40 nmol) in 200 μL of acetone 1 h before UVB (0.18 J/cm^2^). The experiment was repeated three times a week for 15 weeks. The results showed that luteolin inhibited UVB-induced MMP-1 expression, suppressed the activation of AP-1, inhibited JNK1 and p90 RSK2 activity, and additionally, it inhibited wrinkle formation and MMP-13 expression [72]. Heo et al. (2021) conducted a study to test the protective effects of a supplement formula containing lutein, zeaxanthin, and rosemary against skin dehydration due to UV radiation. The study involved 48 male Swiss albino mice aged 8–12 weeks, divided into eight groups of 6 mice each. The groups were as follows: 1. Control group without treatment and without skin shaving; 2. Shaved skin group without treatment; 3. Pathological control group; 4. Standard group treated with hyaluronic acid. 5–8. Treatment groups receiving low and high doses of two different test substances, respectively. Mice in groups 2–8 had their backs shaved and were exposed to UV radiation in the range of 260–400 nm for 15 min daily over 6 weeks. The researchers examined the levels of collagenase, hydroxyproline, hyaluronic acid, ceramides, as well as assessed skin elasticity and water content in the stratum corneum. Based on these parameters, the patented product with lutein was found to ensure high skin protection against skin dehydration caused by UV radiation [51].

### 4.4. β-Carotene

β-carotene is one of the most well-known carotenoids. It is present in various vegetables, including carrots, pumpkins, yams, yellow peppers, and corn. As an antioxidant, it is a powerful free radical scavenger, especially important in maintaining healthy skin. It is also referred to as “provitamin A” because it serves as a precursor of vitamin A. In fact, up to 50% of the consumed β-carotene can be converted to vitamin A. In the intestinal mucosa, β-carotene undergoes enzymatic breakdown by dioxygenase, resulting in the formation of retinal, which is further reduced to retinol, i.e., an active form of vitamin A [57]. Vitamin A is extremely valuable for maintaining youthful and nourished skin, and it also prevents premature aging through its antioxidant and nutritional properties.

Eicker et al. (2003) conducted a study using human foreskin fibroblasts cells that were exposed to UVA radiation (at a power of 70 mW) 3 times a day for 4 days. The cells were supplied with a solution containing β-carotene. The study revealed that β-carotene was absorbed into the cells and interacted with UVA radiation. Furthermore, it provided protection against mtDNA mutation, indicating its potential in preventing photoaging [63]. In another study, eight-week-old male BALB mice were divided into control and test groups. The control group followed a standard diet, while the study group received 100 g of a standard diet supplemented with 50 mg of β-carotene for 3 weeks. After sacrifice, biopsies were taken from the mice’s backs and immersed in saline. The homogenates were then irradiated with a UVA lamp, delivering a dose of 8.21 J/cm^2^. The results demonstrated that β-carotene accumulated in the skin of the study group. In the control group, UVA radiation caused a significant increase in in thiobarbituric acid-reactive substances (TBARS). However, in the study group no increase in TBARS was observed, suggesting that β-carotene supplementation prevented UVA-induced lipid peroxidation [64]. Cho et al. (2010) conducted a study involving 30 healthy women over 50 years of age, who were divided into two groups. For three months, one group received a low dose of 30 mg/day of β-carotene, while the other a high dose of 90 mg/day of β-carotene. The skin of the participants’ buttocks was exposed to UV radiation, and samples were taken for testing. Measurements of wrinkles and skin elasticity were performed at the beginning of the study and after 90 days. The results showed that supplementation with low doses of β-carotene led to an increase in the expression of type I procollagen gene and protein in human skin. Moreover, the supplementation resulted in a reduction of facial wrinkles, improvement in skin elasticity, and a decrease in UV-induced erythema and DNA damage. On the other hand, supplementation with high-dose β-carotene was not associated with significant improvements in signs of cutaneous photoaging and did not confer photoprotection. In fact, it made the skin more susceptible to UV-induced erythema [67]. Lee et al. (2000) also reported that supplementation with natural carotenoids may provide partial protection against UVA and UVB radiation, which can cause skin erythema. The experiment involved 22 participants (11 men and 11 women), who were supplemented with natural carotenoids for 24 weeks. During the first 8 weeks, the subjects were given a daily dose of 30 mg of carotenoids, including 29.4 mg of β-carotene and 0.36 mg of α-carotene in vegetable oil. The amount was increased by 30 mg every 8 weeks, reaching a maximum dose of 90 mg. In order to determine the minimum erythema dose (MED), a 1 cm^2^ area of each participants’ skin was exposed to increasing doses of UV radiation (16–42 mJ/cm^2^). During the supplementation with natural carotenoids, the MED was significantly increased (*p* < 0.05). After 24 weeks of supplementation, serum β-carotene levels rose from 0.22 μg/mL (95% CI; 0.16–0.27) to 1.72 μg/mL (95% CI; 1.61–1.83). Furthermore, the study found a significant inhibition of serum lipid peroxidation (*p* < 0.05) [68].

## 5. Conclusions

Excessive exposure to UV radiation can have numerous detrimental effects on the skin. While using photoprotective preparations is important, paying attention to your diet can also play an important role in preventing photoaging. The active substances found in vegetables have been shown to have a positive impact on the condition of the skin, making it essential to incorporate them into a diet, especially during periods of higher sun exposure such as summer. Vegetables are rich in nutrients that can benefit the skin. Although there are numerous scientific studies focusing on individual vegetables and their bioactive components, there has been a lack of a comprehensive scientific article summarizing the effects of vegetables on skin photoaging. This review aims to address the gap by compiling studies on vegetables that have demonstrated significant effectiveness in photoprotection of the skin. It also highlights the bioactive substances present in these vegetables and their effects. Vegetables such as: broccoli, cucumber, kale, tomato, and carrot, have been found to not only enhance the appearance of the skin, but also to offer protection against UV radiation. Consumption of 40 g of tomato paste per day (containing 16 mg of lycopene) can reduce the changes in UV light-induced erythema by 40% and supplementation with carotenoid-rich natural kale extract (1650 µg carotenoids) can significantly prevent age-related degradation of collagen I in the dermis. Bioactive substances, such as sulforaphane, lycopene, lutein, or β-carotene, which are present in vegetables possess antioxidant properties that counteract the effects of ROS, a major contributor to premature skin aging. This means that maintaining a proper diet that includes these vegetables can aid in preventing skin photoaging, reducing the risk of sunburn, discoloration, and can even help prevent skin cancer.

However, more research particularly in humans, is needed to accurately assess the direct effect of vegetables on the photoaging process.

## Figures and Tables

**Table 1:** The photoprotection effect of natural extracts form vegetables and their active compounds.

Compounds	Type of Extract	Type of Study	Activity	References
Sulphoraphane	Broccoli sprout extracts	In vivo/oral aplicated/ SKH-1 female hairless mice	↓ incidence, number and volume of the tumor ↑ Protection against UV radiation	[28]
	Broccoli sprout extracts (in 80% acetone) containing 1 mmol of 0.3 μmol sulforaphane or carrier	In vitro/ Human HaCaT cells In vivo/topical aplicated/ female SKH-1 hairless mice/mouse PE keratinocytes	↓ number and volume of the tumor ↑ NQO1, glutathione ↑ inhibition of iNOS	[27]
	Broccoli sprout extracts (in 80% acetone:20% water by volume)	In vivo/topical aplicated/ SKH-1 female hairless mice	↑ protein levels of NAD(P)H:quinone ↑ oxidoreductase 1 (NQO1) ↑ glutathione S-transferase A1 ↑ heme oxygenase 1	[29]
	Broccoli sprout extract	In vivo/topical aplicated/ SKH-1 hairless mice	↑ phase 2 enzymes ↑ protect against UVR-induced inflammation and edema in mice ↓ susceptibility to erythema (311-nm UVR) in humans	[33]
	Broccoli sprout extract	In vivo/topical aplicated/ WT and Nrf2–/–C57BL/C57BL/6 male mice	↑ NRF2, ↓ mottled hyperpigmentation of the treated areas ↓ 50% melanin deposition ↓ 30% reduction in the fold change of tyrosinase expression	[31]
	Sulforaphane (synthetic)	In vitro/ dermal fibroblasts or keratinocytes isolated from SKH-1 hairless mice	↑ NQO1 ↓ ROS caused by UV radiation	[62]
	Sulforaphane (synthetic)	In vivo/topical and oral aplicated/ Swiss albino mice	↑ NRF2 ↓ sulfatase-1, 2 ↓ gene and protein expression TNF-α, IL-1β, caspase-3 blocked sulfatase-2 activity ↑ HSPGs, G7 ↓ glypican-3	[35]
	Sulforaphane (synthetic)	In vivo/topical aplicated/ Nrf2 KO, WT C57BL/6 mice	↑ NRF2 ↑ Il-1β, IL-6	[32]
Glucoraphanin	Spray-dried kale and GEK juice (Glucoraphanin)	In vivo/oral aplicated/ male SAMP1 mice	↑ suppressed the thinning of the dorsal skin layer ↑ collagen production a ↑ Nrf2 and HO-1 expression level ↑ TβRII, Smad3 expressions	[53]
Lycopene	Powdered tomatoes (feed with 10% lycopene)	In vivo/oral aplicated/ male and female SKH-1 hairless mice	↑ carotnoids in the skin and plasma ↓ number of emerging tumors ↓ inflammation and subsequent DNA damage in the skin	[49]
	Lycopene in acetone	In vivo/topical aplicated/ SKH-1 hairless mice	↑ inhibition of epidermal ornithine decarboxylase activity ↑ inhibition of andmyeloperoxidase	[50]
Luteolin	Luteolin (98%) was purchased from Indofine Chemical	In vitro/ Human HaCaT cells In vivo/topical aplicated/ SKH-1 hairless mice	↑ inhibition UVB-induced MMP-1 expression in HaCaT cells ↑ inhibition of JNK1 and p90RSK2 activity ↑ inhibition of AP-1	[30]
	Patented supplement (lutein, zeaxanthin and rosemary) dispersed in sunflower oil XanMax^®^ 80	In vivo/topical aplicated/ Swiss albino mice	↑ protective effect skin dehydration due to UV radiation	[51]
B-caroten	Stock solutions of betacarotene	In vitro/ Human fibroblast culture from a human foreskin	↑ protection against mtDNA mutation, which proves the prevention of photoaging ↓ singlet oxygen quencher	[63]
	β-Carotene, β-apo-8V-carotenal, and δ-tocopherol,	In vivo/oral aplicated/ male BALB mice	↑ increase β-carotene in TBARS ↑ prevention UVA-induced lipid peroxidation	[64]

↑—increase; ↓—decrease; GEK: glucoraphanin-enriched kale; UV: ultraviolet; UVA: ultraviolet A; UVB: ultraviolet B; MMP-1: matrix metalloproteinase-1; TBARS: thiobarbituric acid-reactive substances.

**Table 2:** Characteristics of the clinical studies included in this review.

Compounds	Type of Extract	Group Characteristics and Type of Application	Effect	References
Sulphoraphane	Broccoli sprout extracts (in 80% acetone:20% water by volume)	Human subject/ topical aplicated—17 patients, aged 25–51 years	↑ protein levels of NAD(P)H:quinone ↑ oxidoreductase 1 (NQO1) ↑ glutathione S-transferase A1 ↑heme oxygenase 1	[29]
	Broccoli sprout extract	Human subject/ topical applicated—6 patients, aged 28–53 years	↑ phase 2 enzymes ↑ protect against UVR-induced inflammation and edema ↓ susceptibility to erythema (311-nm UVR) in humans	[33]
	Broccoli sprout extract	Human subject/ topical aplicated—7 patients	↑NRF2, ↓ mottled hyperpigmentation of the treated areas ↓ 50% melanin deposition ↓ 30% reduction in the fold change of tyrosinase expression	[31]
Likopen	Tomatoe paste 55 g with 10 g olive oil on white bread	Human subject/ oral aplicated—20 patients, aged 21–47 years	↓ ROS ↓ MMP-1 ↓ mtDNA 3895-bp deletion ↑ procollagen deposition	[65]
	Gel–emulsion containing lycopene, and gel-emulsjon containing Vit. C and E	Human subject/ topical aplicated—10 patients, aged 20–42 years	↑ protective effect against UV-induced erythema	[65]
	Tomatoe puree + olive oil carrot juice lycopene supplement with tomatoe extract Lycopene drink (from tomatoe extract) and synthetic lycopene	Human subject/ oral aplicated—22 patients, aged 26–67 years	↑ protection against UV-induced erythema	[56,59]
	1. Capsules with synthetic lycopene. 2. Tomatoe extract capsule. 3. 250 mL solubilized lycopene drink	Human subject/ oral aplicated—36 patients	↑ protective effect against UV-induced erythema	[66]
B-carotene	Carotene filled in a soft capsule	Human subject/oral aplication—30 patients, over the age of 50 years	↑ expression of type I proctocollagen gene and protein ↓ facial wrinkles ↓r UV-induced erythema and DNA damage	[67]
	Supplementation with 30 mg of natural carotenoids containing 29.4 mg of beta-carotene, 0.36 mg of alpha-carotene, and trace amounts of other carotenoids in vegetable oil	Human subject/ oral aplicated—22 patients,	↑ MED ↑ serum beta-carotene levels ↑ inhibition of serum lipid peroxidation	[68]

UVR: ultraviolet radiation; ROS: reactive oxygen species; UV: ultraviolet; MED: minimum erythema dose.

## Data Availability

Detailed data available from the authors.
